# Novel characteristics of the avian gyrovirus 2 genome

**DOI:** 10.1038/srep41068

**Published:** 2017-02-15

**Authors:** Shuai Yao, Xiang Gao, Tianbei Tuo, Chunyan Han, Yulong Gao, Xiaole Qi, Yanping Zhang, Changjun Liu, Honglei Gao, Yongqiang Wang, Xiaomei Wang

**Affiliations:** 1 Division of Avian Infectious Diseases, State Key Laboratory of Veterinary Biotechnology, Harbin Veterinary Research Institute, Chinese Academy of Agricultural Sciences, Harbin, 150069, China; 2 Northeast Agricultural University, 150030, Harbin, China; 3 Northeast Forestry University, 150040, Harbin, China

## Abstract

Avian gyrovirus 2 (AGV2) was the second member of the viral genus *Cyclovirus* to be discovered. This virus poses a significant potential threat to humans and poultry due to its global dissemination and infectiousness. We used three overlapping polymerase chain reactions (PCRs) to map the whole genome of AGV2. We then modelled the evolutionary history of these novel sequence data in the context of related sequences from GenBank. We analysed the viral protein characteristics of the different phylogenetic groups and explored differences in evolutionary trends between Chinese strains and strains from other countries. We obtained 17 avian-sourced AGV2 whole genomes from different regions of China from 2015 to 2016. Phylogenetic analyses of these Chinese AGV2 sequences and related sequences produced four distinct groups (A–D) with significant bootstrap values. We also built phylogenies using predicted viral protein sequences. We found a potential hypervariable region in VP1 at sites 288–314, and we identified the amino acid changes responsible for the distinct VP2 and VP3 groups. Three new motifs in the AGV2 5′-UTR direct repeat (DR) region were discovered and grouped. The novel characteristics and diverse research on the AGV2 genome provide a valuable framework for additional research.

Avian gyrovirus 2 (AGV2) was first reported by Rijsewijk *et al*. in early 2011 in diseased chickens from Brazil^1^, making it the second member of the viral genus *Cyclovirus* to be discovered. The AGV2 genome consists of ~2.38 kb, with three partially overlapping open reading frames (ORFs): VP1 from nucleotides 953–2335; VP2 from nucleotides 450–1145; and VP3 from nucleotides 577–951. These ORFs encode homologues of the chicken anaemia virus (CAV) viral protein, and these sequences have only ~40% nucleotide similarity with those of CAV^1^. Human gyrovirus (HGyV), which differs from AGV2 by only 3–7% in its VP1–3 protein sequences^2^, was reported in the same year. AGV2 infections have been identified in several different locations in France, Brazil, Italy, Hungary, the Netherlands, South Africa, and Chile, suggesting a worldwide distribution^3^,^4^,^5^,^6^,^7^,^8^. A study focusing on the detection of AGV2 genomes in commercially available poultry vaccines showed that AGV2 genomes were mixed in 9/32 live vaccines produced in Brazil, Canada, and the Netherlands^9^. It was reported that a breakout of the coinfection of AGV2 with avirulent Newcastle disease virus (NDV) caused neurologic symptoms and high mortalities in South Africa^7^. Moreover, PCR assays by Biagini^3^ and Maggi^5^ were positive for HGyV/AGV2 DNA in the blood of healthy humans, transplant patients and HIV-positive patients. Thus, the widespread infection of AGV2 has recently drawn significant attention.

In 2015, the virus was detected for the first time in a chicken flock and healthy humans in mainland China^10^. To explore the prevalence and molecular characteristics of AGV2, an epidemiologic investigation in several regions of China conducted by our group showed that AGV2 was detected with a positive rate of 12.28% from April 2015 to April 2016^11^. However, the molecular characteristics and diverse research on AGV2 have been unreported until the present.

## Results

### Whole genome analysis and grouping of AGV2

We obtained 17 avian-sourced AGV2 complete genomes, each of which had a length of 2376 bp. These sequences had ~40.54% nucleotide similarity with CAV strain Cux-1 (M55918). However, they had very high levels, 93.8–99.3%, of nucleotide similarity with all six AGV2 whole genomes from GenBank (HM590588, FR823283, JQ308212, KJ452213, KJ452214, and KU168250) and much higher similarities with each other (95.7–100%). Sequences of the virus were named using the format PPXXXX, where PP is the area and XXXX is the time of origin. We appended a suffix sequence number if further naming resolution was necessary. Sequences were deposited in GenBank under accession numbers KX708506–KX708522 for whole genomes.

There were 179 mutations in the AGV2 whole genomes, of which 143 were found in more than one sequence (data not shown); the change rate was 7.53%. These mutations changed the number of important *Hind* III and *Bam* HI endonuclease sites, suggesting evolution within the population. Eight sequences (HLJ1508, HE1511, JL1511, JX1602, HLJ1603-1, HLJ1603-2, HM590588, and JQ690763) had one *Hind* III site and two *Bam* HI sites; eleven sequences (HLJ1510, NX1506-1, NX1506-2, JL1508, BJ1509, NX1510, LN1511, GS1512, GZ1601, HLJ1506-2, and KJ452214) had two *Hind* III sites and two *Bam* HI sites; two sequences (HLJ1506-1 and KJ452213) had no *Hind* III and two *Bam* HI sites; and two sequences (FR823283 and KU168250) had two *Hind* III sites and one *Bam* HI site.

**Figure 1:**
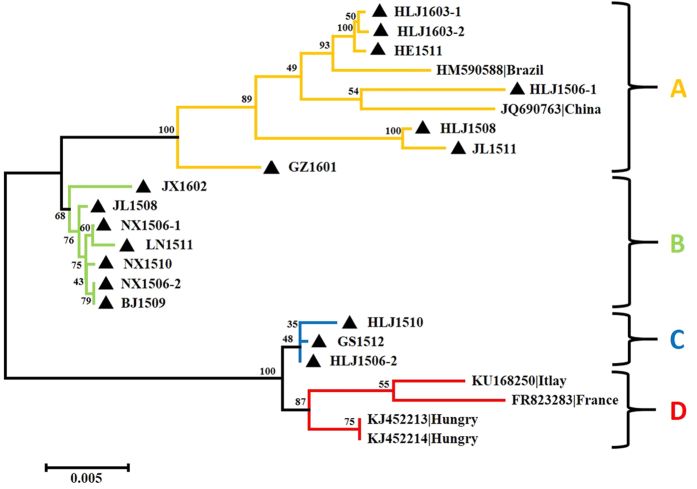
Phylogenetic analysis of the nucleotide sequences of 17 new complete genomes from China and all 6 particular relevant sequences currently available in GenBank. Sequences from the present study (black closed triangles) are named as previously mentioned. Sequences from GenBank were given the country name followed by accession number. The four major groups were identified as A, B, C, and D in yellow, green, blue, and red, respectively. The percentages of replicate trees in which the associated taxa are clustered together in the bootstrap test (1000 replicates) are shown next to the branches.

Phylogenetic analysis at the nucleotide level of the 17 avian-sourced AGV2 genomes sequenced in this study, together with that of all six (de-duplication) AGV2 and HGyV strains from different sources including the chicken (HM590588, Brazil; KU168250, Italy), human (FR823283 France; JQ690763 China) and ferret (KJ452213 Hungary; KJ452214 Hungary), revealed four distinct sequence groups (A, B, C, and D), with bootstrap values of 100, 68, 100 and 87, respectively. The sequences in this study were the majority of the members in Groups A, B, and C (Fig. 1). Group A consisted of nine (39.13%) genomes that originated from China and Brazil; seven members were new AGV2 sequences, mainly from Northern China (HLJ1603-1, HLJ1603-2, HE1511, HLJ1506-1, HLJ1508, JL1511, and GZ1601). Seven (30.43%) highly similar new sequences (JX1602, JL1508, NX1506-1, LN1511, NX1510, NX1506-2, and BJ1509) constituted Group B, which contained no other strains. Three (13.04%) sequences (HLJ1510, GS1512, and HLJ1506-2) constituted Group C, and Group D consisted of four (17.39%) reference sequences from Europe (Fig. 1).

### Grouping and analysis of AGV2 VP1 amino acids and a suspected hypervariable region

**Figure 2:**
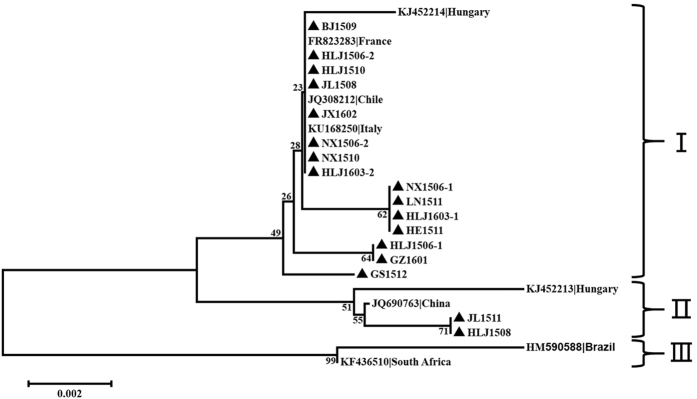
Phylogenetic analysis of the amino acid sequences of 17 new VP1 sequences from different provinces in China and all 8 relevant sequences (6 from complete genomes, one from complete CDS, and one from partial CDS) currently available in GenBank. Sequences in this study (black closed triangles) were named as previously mentioned. Sequences from GenBank were given the country name followed by accession number. The percentages of replicate trees in which the associated taxa are clustered together in the bootstrap test (1000 replicates) are shown next to the branches. The three major groups were identified as I, II, and III.

All VP1 sequences reported presently and obtained in this study are 1383 bp in length. Based on the multiple alignments of these DNA sequences, the maximum divergence is 7.3%. An alignment of the predicted amino acid sequences yielded a maximum divergence of 2.7%. Three major groups were present in the VP1 protein sequence phylogenetic tree. Most of the virus sequences in this study were clustered in Group I, with three European sequences (KJ452214, FR823283, and KU168250) and an avian-sourced sequence from Chile (JQ308212). Group II contained HLJ1508 and JL1511, one reference sequence from China (JQ690763), and one from Hungary (KJ452213). Group III contained two sequences: the first-discovered prototypic AGV2 from Brazil (HM590588) and another avian-sourced sequence from South Africa (KF436510) (Fig. 2). Some protein sequences in Group I (BJ1509, HLJ1510, JL1508, JX1602, NX1506-2, and NX1510) were isolate capsids in Group B of the whole genome (Fig. 1), and those in Group II were all isolate capsids of the whole genome of Group A.

Substitutions at certain positions determined the phylogenetic groupings of this virus protein. Minority characters at VP1 sites 154 (A to S), 288 (V to Q), and 242 (G to Q) belonged to Group II sequences. Substitutions at sites 212 (K to R), 242 (R to G), 270 (S to A), 310 (E to Q), 383 (P to Q), 401 (V to M), and 416 (L to I) were specific to sequences HM590588 and KF436510 of Group III.

**Figure 3:**
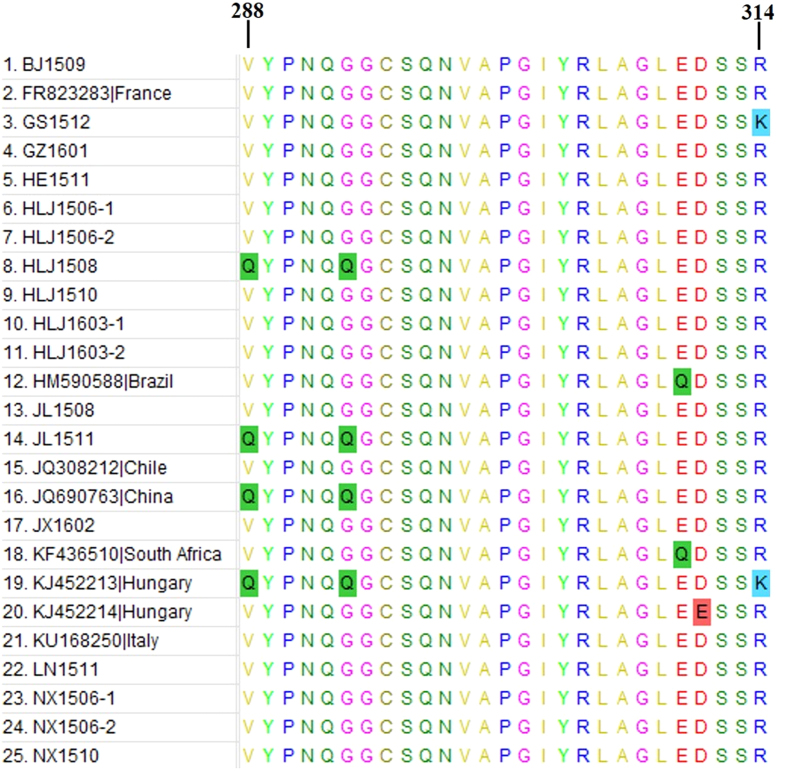
Amino acid alignments of suspected highly variable regions of different AGV2 VP1 coding sequences (aa 288 to 314). Sequences are compared with each other, and amino acids are indicated by a single-letter code. The differences are highlighted by the colour of the background. Nucleotide substitutes occur at sites 288, 293, 310, 311, and 314.

There were 15 amino acid substitutions in the VP1 protein (amino acid sites 36, 95, 154, 212, 242, 270, 288, 293, 310, 311 314, 383, 401, 416, and 459), which on average is one substitution for approximately every 31 amino acids. We suspect that a hypervariable region is present in the middle and posterior of the VP1 protein, from sites 288 to 314. This region has 5 substitutions over its 26 amino acid length, which may be comparable to the CAV hypervariable region (sites 131–165)^12^ (Fig. 3).

### Conservatism and unique amino acids in AGV2 VP2

**Figure 4:**
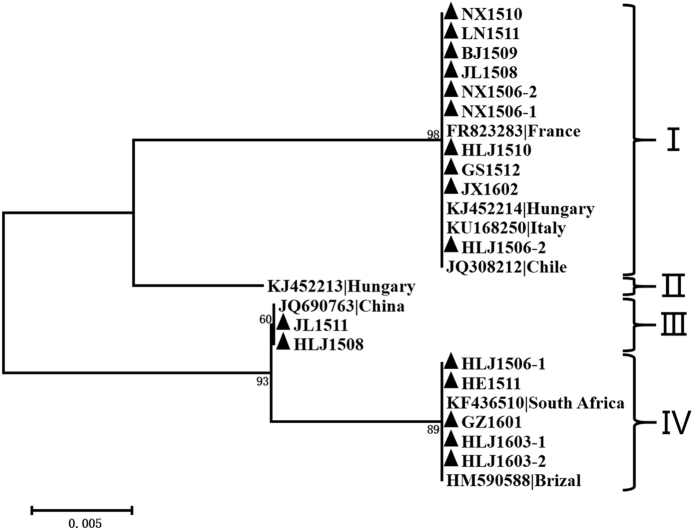
Phylogenetic analysis of the amino acid sequences of 17 new VP2 sequences from different provinces and all 8 relevant sequences (6 from complete genomes and 2 from complete CDS) currently available in GenBank. Sequences in this study (black closed triangles) were named as previously mentioned. Sequences from GenBank were given the country name followed by the accession number. The percentages of replicate trees in which the associated taxa are clustered together in the bootstrap test (1000 replicates) are shown next to the branches. The four major groups of VP2 were identified as I, II, III, and IV.

The VP2 sequences reported presently and obtained in this study are 696 bp in length, except for the ferret-sourced sequence KJ452213, which has a serine (S) insertion at site 162. The phylogenetic tree of VP2 at the amino acid level has four major groups. Unlike VP1, the VP2 sequences are well conserved within their major phylogenetic clusters; however, the inter-phylum branch lengths are relatively high (Fig. 4). The VP2 sequences in Group IV were all expressed by the isolates whose whole genomes were in Group A (Fig. 1), and the isolates in Group B that expressed VP2 were almost all in Group I.

**Figure 5:**
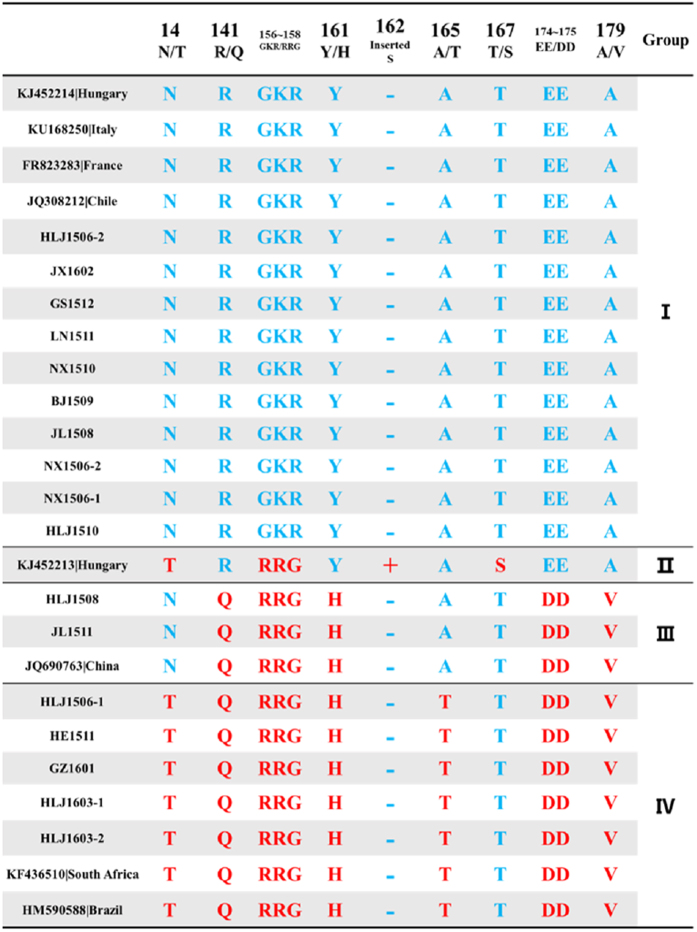
Logical changes in amino acid sequences of VP2. Sequence in this study at the left named as previously mentioned; at the top of this figure, the amino acid sites and the possible amino acids indicated by a single-letter code are displayed. The majority and the minority of amino acid(s) were coloured blue and red, respectively. The symbol ‘+’ represents the insertion, and ‘−’ represents no.

The majority of the same strains (HLJ1506-2, JX1602, GS1512, LN1511, NX1510, BJ1509, JL1508, NX1506-1, NX1506-2, HLJ1510, KJ452214, KU168250, FR823283, and JQ308212) constitute Group I. Substitutions at AGV2 VP2 sites 141 (R to Q), 156–158 (GKR to RRG), 161 (Y to H), 174–175 (EE to DD), and 179 (A to V) characterized Group III, which contained 2 new sequences (HLJ1508 and JL1511) and 1 reference sequence (JQ690763). Group IV contained 5 new sequences (HLJ1506-1, HE1511, GZ1601, HLJ1603-1, and HLJ1603-2) and 2 reference sequences (KF436510 and HM590588) and had nearly the same substitutions as Group III, with additional substitutions at sites 14 (N to T) and 165 (A to T). Group II contained a unique strain (KJ452213) that had a T to S substitution at site 167 and a serine (S) insertion at site 162 (Fig. 5).

### Characterization and grouping of AGV2 VP3 amino acids

**Figure 6:**
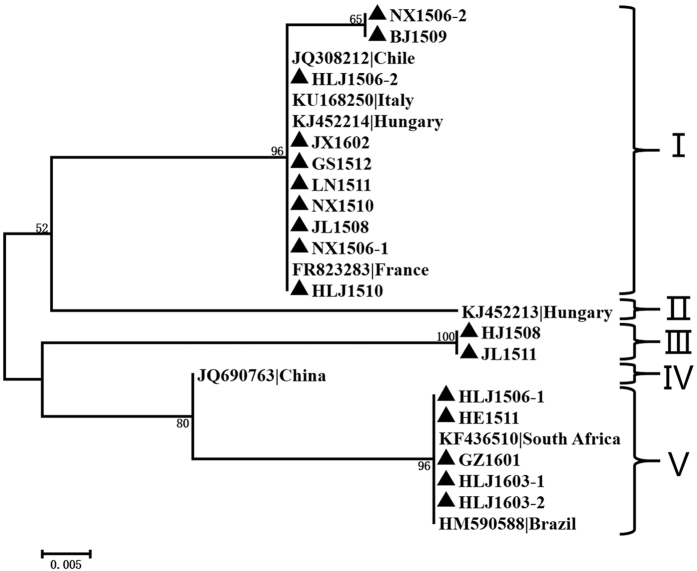
Phylogenetic analysis of the amino acid sequences of 17 new complete VP3 sequences from different provinces and all 8 relevant sequences (6 from complete genomes and 2 from complete CDS) currently available in GenBank. Sequences in this study (black closed triangles) were named as previously mentioned. Sequences from GenBank were given the country name followed by the accession number. The percentages of replicate trees in which the associated taxa are clustered together in the bootstrap test (1000 replicates) are shown next to the branches. The five major groups of VP3 were identified as I, II, III, IV, and V.

Almost all the novel VP3 predicted protein sequences were 124 amino acids in length, except for the unique sequence KJ452213, which has an arginine (R) insertion at site 122. Based on their bootstrap values, the VP3 sequences fell into five groups (Fig. 6). The VP3 expressed by the isolates in the whole genome Group B were all in Group I (Fig. 1), and the isolates in Group A of the whole genome corresponded to those with VP3s in Group V.

The amino acid variations observed in the sequences occurred at 9 significant sites (9, 14, 28, 79, 81, 99, 103, 104, and 115) at both the N- and C-termini, and two amino acid orders at these sites were the basis of the grouping. All fourteen VP3 members of Group I contained the homologous sequence R-Q-S-V-L-A-K-Q-N (NX1506-2, BJ1509, HLJ1506-2, JX1602, GS1512, LN1511, NX1510, JL1508, NX1506-1, HLJ1510, JQ308212, KU168250, KJ452214, and FR823283). Group V contained two reference sequences and five new VP3 proteins with the sequence Q-R-C-A-S-S-R-R-E (KF436510, HM590588, HLJ1506-1, HE1511, GZ1601, HLJ1603-1, and HLJ1603-2), but other sequences did not follow this pattern.

### New pattern in motif of direct repeat region in AGV2 5′-UTR

**Figure 7:**
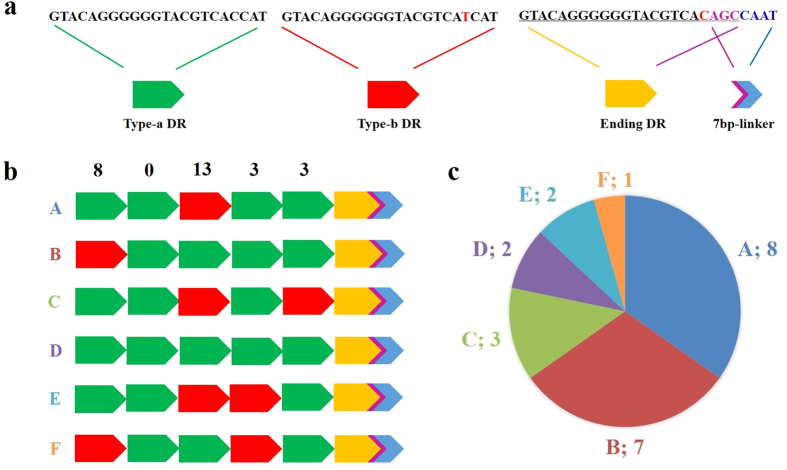
Different structures, combinations and appearance rates of DR regions in AGV2. (**a**) Nucleotide sequences of the three types of DR. Type-a DR is covered by green; Type-b DR is covered by red and has a substitute at the 19th base of Type-a DR highlighted in red; Ending DR is covered by yellow, with the nucleotide sequence underlined; the 7-bp linker is partially covered by blue; and other overlapping with the Ending DR is covered by violet. (**b**) Different combinations of DR region. The graphics represent different DRs, as described in (**a**). Each combination was grouped as A, B, C, D, E, or F and was differently coloured. Type-b DR as each component of the DR combination is displayed above the component column. (**c**) Proportion of each group of DR combinations. The number of each group is shown in the pie chart.

The 5′-untranscribed region (5′-UTR) of AGV2 is by definition located between the canonical polyadenylation site (AATAAA) and the start of transcription^1^. Though the function of the noncoding region is not yet known, it may have the same promoter-enhancer region as its CAV counterpart, which includes four or five 21-base direct repeats (DR) and an indispensable 12-bp insert^13^,^14^,^15^. The 5′-UTR of AGV2 contained 6 continuous DRs without an inserting fragment, and these AGV2 DRs were each 22 bases in length. We observed three different types of DR sequence. The majority of these DRs were defined as Type-a DRs of the nucleic acid sequence 5′-GTACAGGGGGGTACGTCACCAT-3′, while the minority Type-b DR had a mutation at the 19^th^ Type-a base (C to T) as follows: 5′-GTACAGGGGGGTACGTCATCAT-3′. The Ending DR was the last DR in the string, and it differed at its 3′ terminus from the other DR types: 5′-GTACAGGGGGGTACGTCACAGC-3′. The last four bases of the Ending DR form the first half of a 7-bp linker: 5′-AGCCAAT-3′ (Fig. 7a).

Based on the structural characterization of the AGV2 repeat units, six groups of DR region were classified. The combination of Type-b DR occupies the third and the first places and is defined as Group A and Group B, respectively. Group E is the combination of two Type-b DRs occupying the third and fourth places. Groups C, D, and F were three novel groups discovered in this research. The newfound Group C had two Type-b DRs as the third and fifth components, yet Group D had no Type-b DR and Group F had two Type-b DRs occupying the first and fifth places (Fig. 7b).

Group A (8, 34.78%) and Group B (7, 30.43%) made up more than half of all DR regions, while Group E (2, 8.70%) and the three novel groups (C, D, and F) made up lower percentages (Fig. 7c). The number of Type-b DRs are displayed above each column; altogether, eight, zero, thirteen, three, and three Type-b DRs are the total numbers of each component shown in Fig. 7b. However, Type-a DRs made up the vast majority (79.23%, 103/130) of substitutable components in the DR combinations, unlike the third component, in which Type-b held more than half of the seats (13/23) (Fig. 7b).

## Discussion

This report is the first to characterize AGV2 genomes originating from diverse geographic locales around the world and different hosts, and it provides significant additional AGV2 genomic information for the six unique complete genomes, one complete coding sequence (CDS) and one partial CDS previously retrievable from GenBank. This study provides a basis for further research on the relationships between the genetic element structure and virus phenotype.

The complete genomes that we report here are each 2376 bp in length, while all of the other sequences in GenBank are approximately 2380 bp in length. The length of the poly C and G regions upstream of the 5′-UTR account for this discrepancy. The phylogenetic analysis of the CDS at the amino acid level did not perfectly match the groupings based on the nucleotide sequences. This mismatch may be due to synonymous mutations; AGV2 isolates should be almost identical at the amino acid level, despite differences at the nucleotide level.

All the sequences that we obtained were highly similar. The pairs NX1506-2 and BJ1509 and HLJ1506-2 and GS1512 had particularly high pairwise similarity, but entirely different backgrounds. The same sequences were detected at different times and places, meaning that they may be epidemic strains in China or that some transmission carried them from one place to another during this time. The vaccine may be a reason for the observed similarities, in light of the role of AGV2 as a contaminant in poultry vaccines^9^. Alternatively, the strains with very similar backgrounds were not fully consistent. HLJ1506-1 and HLJ1506-2 shared 96% homology at the nucleotide level and belonged to different branches of the phylogenetic tree (Fig. 1). Hence, at least two AGV2 strains were circulating within the same time and place. However, the pair NX1506-1 and NX1506-2 and the pair HLJ1603-1 and HLJ1603-2 each had two mutations. These two pairs had one common mutation at nucleotide 2328, which substituted the penultimate amino acid of VP1 (threonine for asparagine). Altogether, four sequences in the present study had this mutation. The two pairs each additionally had one other mutation. The mutation at nucleotide 611 of NX1506-1 and NX1506-2 caused an amino acid substitution in the N-terminal of the 12^th^ amino acid of VP3 (threonine in NX1506-1 and isoleucine in NX1506-2). The other mutation at nucleotide 157 of the 5′-UTR in HLJ1603-1 and HLJ1603-2 was between a cytosine and a thymine. These observations indicate that these two couples are the same strains with a few mutations, and the consequences of these relationships merit further investigation.

The epidemic situation of AGV2 varies throughout the world. The sequences in this study had similar substitutions as variants from southern Brazil and the Netherlands^4^ but different change trends. The majority and minority amino acid of the new sequences were Y&H, respectively, at site 161 in VP2 and V&A, L&S, A&S, R&K, K&R, Q&R, and N&E, respectively, at sites 79, 81, 85, 99, 101, 104, and 115 in VP3, which was the exact opposite of the results from southern Brazil and the Netherlands. It can be inferred that the primary epidemic strains had regional differences. The Brazilian strains had substitutions at sites 113 and 120 of VP3, which were R to K and K to R. The possibility that these differences are related to the high incidence of AGV2 in Brazil warrants further study.

The study of the virus capsid protein VP1 may yield virus information related to the receptors and neutralization of the antigenic epitope, as occurred for CAV^16^, which determines cell tropism and immune escape. However, lacking a number of sequences, though not too many mutations in the suspected hypervariable region of VP1, yet illogical changes suggested the trend of diversity. As more AGV2 sequences are reported, the hypervariable characteristics may become more convincing. Three replication motifs were found in VP1 of AGV2: FAALS (sites 325–329), RRWLTLV (sites 363–369) and KAMA (sites 412–415)^1^. These sites were completely conserved among all sequences presently reported.

AGV2 VP2 may have a function similar to CAV VP2. The protein phosphatase activity of CAV VP2 is important but is not absolutely required for replication^17^; however, other mutations, which are expected to have subtle or no effects on the protein phosphatase activity, resulted in varying degrees of impairment to the CAV replication^18^. In contrast to wild-type CAV, VP3 was cytoplasmic rather than nuclear in location in cells infected with VP2 mutants, suggesting a role for VP2 in VP3 trafficking and function^19^. In this study, the phosphatase motif was found to be highly conserved in the sequence WLRQCARSHDEICTCGRWRSH (sites 95–115)^1^, indicating the importance of VP2 for AGV2. Whether and how the other mutations in AGV2 VP2 affect its other functions requires further investigation.

Some important functional domains were predicted in AGV2 VP3, such as apoptin, which is known to translocate to the nucleus in transformed cells; this domain plays a role in the induction of apoptosis in these cells^20^,^21^. A putative LRS (leucine-rich domain) of IQIGIGSTIITLSL at residues 38–51, a putative NES (nuclear export signal) of EKQQKENLI at residues 102–110, a putative NLS1 (nuclear localization signal) of RRPRR at residues 84–88, an NLS2 of GPPIKKLRL at residues 116–124, and a predicted phosphorylation site at threonine 111^22^ have been proposed. The limited mutations in VP3 might not affect its function, except for the NES. Further study on whether or how the substitutions at sites 99, 101, 103, and 104 affect the subcellular distribution and function of AVG2 VP3 is underway by our group.

The 5′-UTR must be important for gene expression and regulation in AGV2. Although the detailed function of the DR region in 5′-UTR is unknown, the disruption of the relative spacing of the DR region with other promoter elements and the start of transcription by the insertion of a 7-bp linker decreases the rate of virus replication in cultures of CAV^14^. It seems probable that the reported strains may have different transcriptional activities or degrees of virulence. Although it is difficult to draw a conclusion from the present data on which DR type was the original, it seems that Type-b DR, as the primary third component with no seat in the second, is not accidental. It can thus be inferred that the type of DR in the second position may be the most influential to the AGV2 life cycle.

In this study, a high homology of AGV2 was observed within different hosts, including chicken, human and ferret, on the nucleic acid and protein levels. One possibility is that not enough various-sourced sequences could be referred; another potential reason is that AGV2 might infect different hosts.

This study provides a basis for future research on the pathogenicity and genomic function of AGV2. We are dedicated to further unveiling the virology of AGV2 and *Cyclovirus*.

## Materials and Methods

### Ethics statement

All applicable international, national, and/or institutional guidelines for the care and use of animals were followed. The animal experiments were performed in strict compliance with the Guideline for the Care and Use of Laboratory Animals of the Ministry of Science and Technology of the People’s Republic of China. The Committee of the Ethics of Animal Experiments at the HVRI, CAAS, also approved the animal experiment protocols.

### Sample information

In the epidemiologic investigation from April 2015 to April 2016 by our group, 55 of 448 clinical specimens from suspected sick fowl or fowl embryos (mostly comprising livers, spleens, and thymuses) tested positive *via* specific PCR^11^. We selected 17 of these positive samples for study based on their diversity of temporal and spatial characteristics; they were collected from several regions of China, including Heilongjiang Province, Ningxia Province, Jilin Province, Beijing, Hebei Province, Liaoning Province, Guizhou Province, and Jiangxi Province, between June 2015 and March 2016. These sequences can be considered to represent the characteristics of a molecular epidemic in China.

### DNA extraction

Clinical samples were oscillated and broken to obtain tissue homogenates as follows. Then, 300 mg of tissues was put into a 2-mL Ep tube with 500 μL of PBS and two high-pressure steam sterilization small steel balls were added, and then the solution was oscillated for 3 min twice to break the tissues at a frequency of 29 Hz (MM400, Restch^®^). Total DNA was extracted from the tissue homogenates as follows. One hundred microliters of tissue homogenates was lysed in 200 μL of tissue lysis buffer (4 M guanidine hydrochloride, 25 mM sodium citrate, and 1% Triton X-100 and extracted twice with phenol-chloroform-isoamyl alcohol (25:24:1). The DNA was precipitated with absolute ethanol, washed with 70% ethanol, and dried at room temperature. Subsequently, the DNA was resuspended in nuclease-free water and stored at −70 °C.

### Amplification of AGV2 genome

Three overlapped PCRs were used to map the AGV2 whole genome. The primers QC2F, 5′-TCA CAG CCA ATC AGA ATT GAG CAC G-3′, and QC2R, 5′-TTC TAC GCG CAT ATC GAA ATT TAC C-3′, were used to amplify the first segment of the genome, covering a 733-bp region from nucleotides 349–1082. The primers QC3F, 5′-TAT TCC CGG AGG GGT AAA TTT CGA T-3′, and QC3R, 5′-CCC CTG TCC CCG TGA TGG AAT GTT T-3′ were used to amplify the second segment of the genome, covering a 981-bp region from nucleotides 1046–2027. The primers QC1F, 5′-ATT TCC TAG CAC TCA AAA ACC CAT T-3′, and QC1R, 5′-TCT GGG CGT GCT CAA TTC TGA TT-3′ were used to amplify the third segment, covering an 802-bp region from nucleotides 1960–379. We based our PCR primers on the reference complete genome sequence HM_590588. The PCR amplification was carried out in PCR buffer, which contained 0.4 mM of each dNTP, 10 pmol of each primer, and PrimeSTAR Max DNA polymerase (TaKaRa Biotechnology Co., Ltd., Dalian, China) in a 50-μL total reaction volume, using an automated thermal cycler (Gene Amp PCR System TC-960F, Blue Marlin, Zurich, Switzerland). These reactions ran under the following program: initial denaturation of 98 °C for 5 min, followed by 30 cycles of denaturation at 98 °C for 10 s, annealing at 60 °C for 15 s, and extension at 72 °C for 10 s; the final extension was carried out at 72 °C for 7 min. The PCR products were analysed by 1.5% agarose gel electrophoresis, and imaged using a BIO-RAD Universal Hood II system. In all PCR reactions, an AGV2 isolate previously identified in our laboratory was used as a positive control, and the PCR mixture was used as a negative control. The 3 regions were purified using an agarose gel with the AxyPrep™ DNA Gel Extraction Kit (AXYGEN). DNA extraction and PCR were performed at least twice for each sample.

### DNA cloning and sequencing

In the case of sequencing failure, the specific PCR products of the 3 regions were subcloned into the pMD18^®^-T Easy vector (TaKaRa Biotechnology Co., Ltd., Dalian, China), and the ligated products were transformed into *Escherichia coli* subcloning efficiency DH5a competent cells (TIANGEN BIOTECH CO., LTD., Beijing, China). Colonies that contained DNA inserts of the correct size were picked and grown overnight in 3 mL of ampicillin-containing lysogeny broth (LB) liquid medium. The mini-preparation of plasmid DNAs was performed with a plasmid extraction kit (AxyPrep™ Plasmid Miniprep, Union City, USA), following the manufacturer’s instructions. The plasmid DNAs were sequenced by Comate Bioscience Co., Ltd. (Changchun, Jilin, China).

### Analysis of sequence data

Contig assembly was performed using Seqman in Lasegene (DNASTAR Inc., Madison, Wisconsin). Sequences were aligned using ClustalW^23^. Phylogenetic and molecular evolutionary analyses were based on the VP1, VP2, VP3, and UTR DNA sequences, using MEGA version 7.0.18^24^. Phylogenetic analyses of the nucleic acid and deduced amino acid sequences were conducted using the neighbour-joining method^25^, the Kimura 2-parameter model^26^, pairwise deletion, and 1000 bootstrap replicates^27^. Relevant whole genome and CDS available on GenBank were included for comparison (Table 1).

## Additional Information

**How to cite this article:** Yao, S. *et al*. Novel characteristics of the avian gyrovirus 2 genome. *Sci. Rep.* **7**, 41068; doi: 10.1038/srep41068 (2017).

**Publisher's note:** Springer Nature remains neutral with regard to jurisdictional claims in published maps and institutional affiliations.

## Figures and Tables

**Table 1:** The sequence information used in this article.

Isolate Name	Time	Location	Source	Accession number
HLJ1506-1▲	Jun-2015	Tsitsihar, HLJ	Chicken	KX708506
HLJ1506-2▲	Jun-2015	Yichun, HLJ	Chicken	KX708522
NX1506-1▲	Jun-2015	Dingbian, NX	Chicken	KX708508
NX1506-2▲	Jun-2015	Zhongwei, NX	Chicken	KX708509
HLJ1508▲	Aug-2015	Harbin, HLJ	Chicken	KX708510
JL1508▲	Aug-2015	Baishan, JL	Chicken	KX708511
BJ1509▲	Sep-2015	Yukou, BJ	Chicken	KX708512
HLJ1510▲	Oct-2015	Mudanjiang, HLJ	Chicken	KX708507
NX1510▲	Oct-2015	Shizuishan, NX	Chicken	KX708513
HE1511▲	Nov-2015	Shijiazhuang, HE	Chicken	KX708514
LN1511▲	Nov-2015	Dalian, LN	Chicken	KX708515
JL1511▲	Nov-2015	Dehui, JL	Chicken	KX708516
GS1512▲	Dec-2015	Uncertainty, GS	Chicken	KX708517
GZ1601▲	Jan-2016	Uncertainty, GZ	Chicken	KX708518
JX1602▲	Feb-2016	Fuzhou, JX	Chicken	KX708519
HLJ1603-1▲	Mar-2016	Jixi, HLJ	Chicken	KX708520
HLJ1603-2▲	Mar-2016	Jixi, HLJ	Chicken	KX708521
915 F 06 007 FD	May-2009	France	Human	FR823283
G13	2011	Hungary	Ferret	KJ452214
G17	2011	Hungary	Ferret	KJ452213
China	2012	China	Human	JQ690763
CL33^a^	2008	Chile	Human	JQ308212
Ave 3	Jul-2006	Brazil	Chicken	HM590588
S53/It	Jan-2014	Italy	Chicken	KU168250
UP455/13^b^	Mar-2013	South Africa	Chicken	KF436510

▲ Obtained in this research.

^a^Complete CDS.

^b^Partial CDS.
